# Pituitary tumorous hyperplasia due to primary hypothyroidism

**DOI:** 10.1007/s00701-012-1342-0

**Published:** 2012-04-17

**Authors:** Lin Han, Junwen Wang, Kai Shu, Ting Lei

**Affiliations:** 1 Department of Neurosurgery, Tongji Hospital, Tongji Medical College, Huazhong University of Science and Technology, Wuhan, China 430030; 2 Department of Neurosurgery, Center Hospital of Wuhan City, Wuhan, China 430014

**Keywords:** Hypothyroidism, Pituitary, Tumorous hyperplasia

## Abstract

**Background:**

To study the diagnostic and therapeutic features of pituitary tumorous hyperplasia due to primary hypothyroidism.

**Methods:**

Fifteen patients with pituitary tumorous hyperplasia were studied in clinical manifestation, pathologic, endocrinological, radiographic and therapeutic features retrospectively.

**Results:**

All of these patients suffered from primary hypothyroidism. Magnetic resonance imaging (MRI) scanning found that there were masses in the sellar region with equal T1 and little longer T2 signal, and which could be obviously enhanced by gadolinium EDTA injection. Diameters of these masses were between 1.1 and 2.5 cm. Thyroxine substitution therapy was ordered. Four months later, MRI scanning found that the masses disappeared and only normal pituitary gland left. Plasma thyroxine, thyroid-stimulating hormone (TSH), and prolactin (PRL) levels dropped to their normal ranges.

**Conclusions:**

Thyroxine substitution therapy was the first choice of pituitary tumorous hyperplasia due to primary hypothyroidism. If they are followed by TSH adenoma, or the optic chiasma was pressed by the enlarged pituitary, transsphenoidal microsurgery could be applied.

Pituitary tumorous hyperplasia due to primary hypothyroidism refers to the enlargement of relevant pituitary-hormone secreting cells, caused by primary target gland hypofunction [1, 8]. Pituitary tumorous hyperplasia due to primary hypothyroidism occurs most frequently among all feedback tumors, occupying 33.3% [3]. Clinical doctors always pay little attention to it, because it can not be differentiated easily from primary thyrotropic adenoma by radiographic features only. However, the therapeutic principles to treat them are completely different, and clinical doctors frequently misdiagnose it and order erroneous treatment. From January 1999 to January 2006, 15 patients with pituitary tumorous hyperplasia due to primary hypothyroidism were hospitalized in our department. The clinical manifestation, pathologic, endocrinological, radiographic and therapeutic features of these cases were studied, analysed and summarized in this paper.

## Clinical data

### General data

There were 15 patients in total with pituitary tumorous hyperplasia due to primary hypothyroidism treated in our department form January 1999 to January 2006. Six of them were males, nine of them were females. The ages of these patients were between 13 and 46 years old (average, 24 years old). The time course of the disease was between 5 months to 3 years (average, 8 months).

### Clinical symptoms

All of the 15 patients had longitudinal symptoms of hypothyroidism, such as acratia, aversion to cold, anorexia, facial edema, pretibial myxedema and so on. Three of them, who were younger than 16 years old, had symptoms of hypoevolutism, including growth stasis, microsoma, and hypogonadism. Seven adult female patients had symptoms of amenorrhea and three of them also had symptoms of lactorrhea.

### Results of laboratory examination

All of the 15 patients had different degrees of hormone disorders. The plasma thyroxines (FT3, FT4) of these patients were below the normal levels. Plasma thyroid-stimulating hormone (TSH) levels in these patients were between 85 and 190 μIU/ml (normal range, 0.38–4.34 μIU/ml), and prolactin (PRL) was between 50.46 and 180.75 ng/ml (normal range, 3.34–26.72 ng/ml). Other pituitary-secreting hormones (GH, ACTH) were in the normal ranges. The concentrations of plasma TG-Ab and TPO-Ab were found to be raised in 11 patients and remained normal in the other four patients. Thirteen of the patients were positive for TMAb.

### Laboratory dynamic examination

The results of thyroid-releasing hormone (TRH) stimulation tests showed enhanced levels in all 15 patients, and the results of TSH stimulation tests were all decreased in these patients.

### Imaging features

**Fig. 1:**
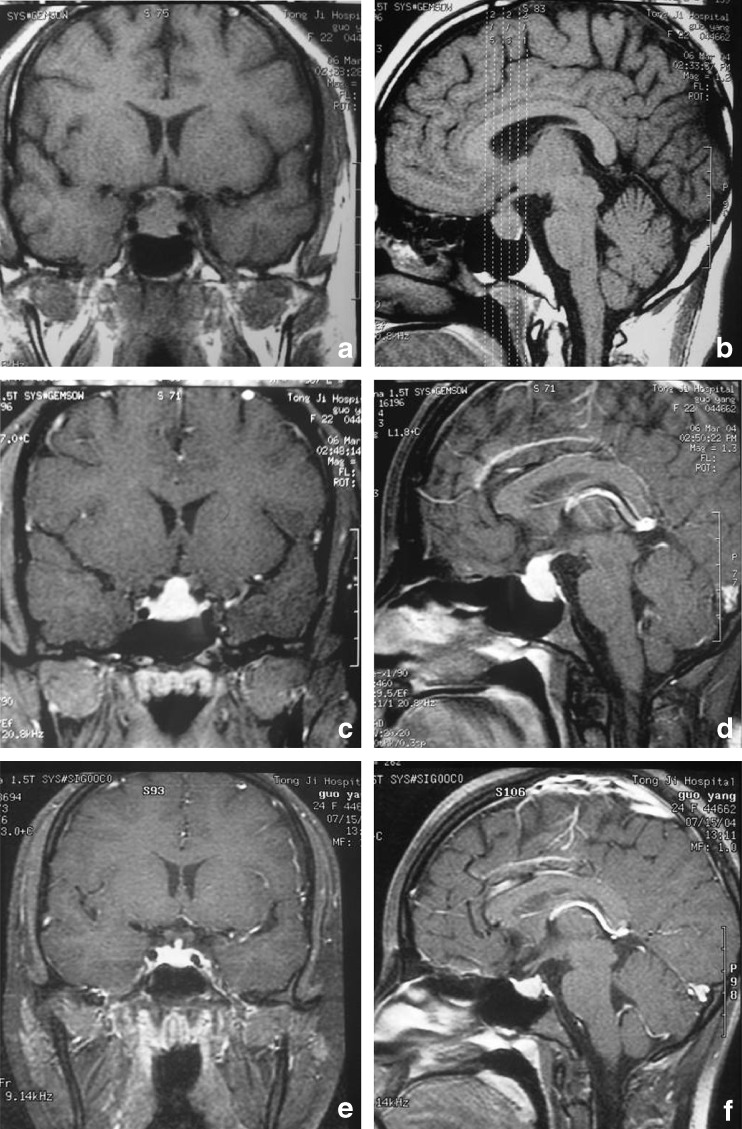
**a**, **b** MRI coronal scan showing pituitary enlargement with equal T1 signal, close to the optic chiasm. **c**, **d** MRI scan showing a pituitary mass obviously and uniformly enhanced by Gd-EDTA injection. **e** and **f** MRI coronal, sagittal scan showing pituitary size return to normal size after taking thyroxine tablets

^99^Tc-nuclide scan was carried out for six patients, which showed heterogeneous absorption decrease. Thyroid color ultrasonography found decreased resonance in six patients, and diffuse heterogeneous resonance in nine patients. Magnetic resonance imaging (MRI) scans were undertaken for 15 patients, and computed tomography (CT) scans for five patients. They all showed a space-occupying mass in the sellar region. The CT scans found an enlarged pituitary, promontory of the superior border, collapse of the saddle floor, thickness and deflection of the pituitary stalk, etc. The MRI scans found a mass in the sellar region with equal T1 and little longer T2 signal, and the mass could be obviously enhanced with Gd-EDTA injection. The diameters of these masses were between 1.1 to 2.5 cm, and some of them grew upward to the suprasellar region and pressed the optic chiasma (Fig. 1a–d).

## Results

All of these hospitalized patients were diagnosed to have primary hypothyroidism according to clinical symptoms, laboratory examinations, laboratory dynamic hormone-secreting examination, and thyroid radiographic examination. The changes in pituitary radiographic features suggested pituitary tumorous hyperplasia due to primary hypothyroidism. All of them were given 80–120 mg thyroid tablets for substitute therapy from low dose to cure dose gradually. The clinical manifestations of hypothyroidism, such as acratia, intolerance of cold, anorexia, facial edema and so on, were relieved obviously. Menstruates in seven patients with amenorrhea and lactorrhea returned to normal cycle. The symptoms of the three with development disorders also were obviously relieved. These patients got more than a 5-cm increase in body height, and return of male genital development or function. Plasma T3, T4, TSH and PRL in these patients returned to the normal range about 30–120 days after therapy (average 85 days). After 4–11 months of substitute therapy, MRI scan found that the enlarged pituitary in these patients returned to normal size (Fig. 1e, f).

## Discussion

Pituitary tumorous hyperplasia due to hypothyroidism is defined by obvious enlargement of the pituitary follicle, hyperplasia of endocrine cells, and changes of pituitary structure. The differences in ultrastructure between hyperplasia cells and normal pituitary cells are very obvious. These are always caused by pathological factors, and these are mostly found in primary hypothyroidism [8].

Primary hypothyroidism is often caused by chronic lymphocytic thyroiditis (also known as autoimmune thyroiditis and Hashimoto’s thyroiditis) [6]. Chronic lymphocytic thyroiditis usually has a deferment disease course with insidious clinical signs and symptoms, and can not be diagnosed and treated timely. Thyroid tissues are infiltrated by multiple lymphocytes and plasma cells in the early stage of chronic lymphocytic thyroiditis, and then the follicular structures are damaged continuously. Dysfunction of thyroid hormone synthesis and secretion appears later. The reduced concentration of serum FT3 and FT4 causes weakness of inhibition to hypothalamus TRH secretion and pituitary TSH secretion through a long loop feedback modulation mechanism, and then the hypothalamus TRH secretion increases, followed by hyperplasia and hypertrophy of pituitary TSH-secreting cells and degeneration of basophilic cells. All of these changes lead to enlargement of anterior pituitary and sometimes adenoma. The increased hypothalamus TRH secretion could not only lead to hyperplasia and hypertrophy of pituitary TSH-secreting cells and increased secretion of TSH but also stimulate PRL-secreting cells, promote PRL synthesis and secretion. Thereby, raised serum PRL level and hyperprolactinemia appear.

Pituitary tumorous hyperplasia due to primary hypothyroidism could not be easily differentiated from primary TSH adenoma, only by radiographic features. But the treatment is obviously different. For primary TSH adenoma, microsurgical treatment is preferred; but for pituitary tumorous hyperplasia due to primary hypothyroidism, thyroxine replacement therapy is preferred [7]. In patients suffering from primary TSH adenoma, symptoms like various degrees of hyperthyroidism and goitre, an increase of serum FT3, FT4, and TSH could be found; but the serum thyroid antibody was negative. However, in patients suffering from pituitary tumorous hyperplasia due to hypothyroidism, decreased serum FT3 and FT4, elevated TSH level can be found. Also, TRH stimulation test showed active results. Sometimes various degrees of hyperprolactinemia could be found.

Substitute therapy is the most important treatment for hypothyroidism [4, 5]. Ozbey et al. [4] once reported the case of a patient suffering from a large pituitary adenoma (which confirmed by pituitary MRI scan) secondary to primary hypothyroidism, hyperprolactinemia and amenorrhea. After treatment with L-T4, plasma T3, T4, TSH, PRL and menstruation returned to their normal ranges, and the large pituitary adenoma disappeared in MRI scan. This prompted that the large pituitary adenoma was caused by the hyperplasia and hypertrophy of TSH and PRL secreting cells. So we could think that thyroxine was the more preferred treatment for patients suffered of primary hypothyroidism with hyperprolactinemia and pituitary enlargement prior to surgery and bromocriptine. Thyroxine substitute treatment showed significant clinical therapeutic effect. The time course of treatment should be more than 4 months and sometimes must be more than 2 years. And if necessary, some patients may need to take the medicine for life. The dosage of thyroxine should begin with a low dose (20–30 mg per day), and increase to maintenance dose gradually every 2–3 weeks. Serum T3, T4, TSH level should be tested regularly during the course of therapy, the dose of thyroxine should be adjusted according to the results, and the treatment should not be stopped, even encountering pregnancy. In this group, all of these patients received substitute thyroxine therapy for 4 months, and then the serum T3, T4, and TSH of these patients returned to their normal ranges. In female patients, menstruation returned and lactorrhea stopped. MRI scan showed intrasellar pituitary “adenoma” shrunk significantly. All of these changes confirmed that the symptoms of lactorrhea, amenorrhea, and pituitary enlargement were attributable to primary hypothyroidism.

Three months after substitute therapy, the repeated test of serum T3, T4, and TSH should be carried out. If the results show that TSH level declined partially, but the thyroid function made no significant improvement, long-term increased secretion of anterior pituitary TSH-secreting cells should be considered [2]; and this may lead to the formation of adenoma on the basis of TSH-secreting cell hyperplasia. If this happened, microsurgical resection via a transsphenoidal approach could be ordered. If the optic nerve or optic chiasm were pressed by the adenoma, microsurgery should be chosen first, to relieve the pressure, and thyroxine tablet substitute therapy should be taken after surgery. If after more than 4–6 months of thyroxine tablet substitute therapy, serum TSH level declined but serum PRL level remained high, and amenorrhea and lactorrhea continued, while enlarged pituitary did not shrink in MRI scan, in this case, we should consider whether there is a pituitary PRL adenoma or not.
